# Research Capacity at Traditional Chinese Medicine (TCM) Centers in China: A Survey of Clinical Investigators

**DOI:** 10.1155/2017/4231680

**Published:** 2017-03-08

**Authors:** Shuo Feng, Mei Han, Lily Lai, Si-cheng Wang, Jian-ping Liu

**Affiliations:** ^1^Center for Evidence-Based Chinese Medicine, Beijing University of Chinese Medicine, 11 North Sanhuan East Road, Chaoyang District, Beijing 100029, China; ^2^Primary Care and Population Sciences, Faculty of Medicine, University of Southampton, Aldermoor Health Centre, Aldermoor Close, Southampton, Hampshire SO16 5ST, UK; ^3^Science and Technology Division, State Administration of Traditional Chinese Medicine of the People's Republic of China, 1 Gongti West Road, Dongcheng District, Beijing 100027, China

## Abstract

*Background.* The development of an evidence-based approach to traditional Chinese medicine (TCM), which depends on the generation of good quality evidence, requires an adequate workforce. However, the research capacity of TCM investigators is not known.* Study Design.* This cross-sectional study was conducted to describe the research capacity of TCM clinical investigators in China.* Participants.* A total of 584 participants from TCM hospitals and research centers were included. They were asked about the academic and research characteristics, needs for research capacity building, and barriers to clinical research.* Results.* The majority (80.82%) were qualified to at least a Master's degree, whilst a smaller proportion (40.24%) held a senior professional title. We found that academic outputs were low with the majority (62.16%) authoring less than five publications in total. The most pressing needs for building research capacity identified were training in research methodology (97.43%) and identification of research questions (86.81%), whilst the highest ranking barriers to conducting research were limited motivation, funding (40.72%), and time (37.15%).* Conclusion.* The methodology training, along with investment in the research workforce, needs to be urgently addressed to improve investigators' research capacity and the development of an evidence-based approach of TCM.

## 1. Introduction

Evidence-based medicine (EBM) has been regarded as the optimal approach to medical practice ever since Sackett et al. introduced the concept in 1996 [1]. EBM as a concept has been widely celebrated and implemented where possible in many countries including China. The World Health Organization (WHO) has advocated a similar evidence-based approach to the practice and evaluation of traditional systems of medicine such as traditional Chinese medicine (TCM) [2]. TCM is used in the treatment and prevention of many conditions in China. Covered by healthcare insurance, TCM is considered integral to China's national health system along with conventional healthcare. Historically, TCM is based on a literature of empirical use but with a growing emphasis on the adoption of evidence-based approach. High-quality research is necessary to inform clinical practice and decision-making in TCM.

In developing countries, the evidence-based medical science is weak in many areas, such as surgery, manual therapy, and oncology [3–5], even as conventional medicine research, which is better funded than TCM research. Despite an unprecedented rise in the number of TCM clinical studies conducted in China in recent years, high risk of bias and serious methodological problems are prevalent amongst TCM studies and systematic reviews published in Chinese [6, 7]. The quality of TCM research is limited by inadequate training, which has an impact on the quality of research products [8, 9]. Moreover, TCM researchers may not be regularly applying the best evidence because of lack of understanding of how to access scientific literature and how to critically appraise published evidence [10–13]. Since 2009, the Chinese government has established 16 TCM clinical research centers throughout China, with the aim of supporting research projects and cultivating clinical investigators, creating better evidence, and informing policymakers, clinicians, and patients. No study to date has reported the status of TCM investigators. We conducted a cross-sectional survey to describe the characteristics of TCM investigators and to identify perceived barriers to, and facilitators for, building research capacity.

## 2. Materials and Methods

### 2.1. Questionnaire Development

The questionnaire was developed in three stages. We performed a literature review by using the search term “research capacity” and which identified 266 publications. Two persons (FS and HM) screened these publications to identify relevant studies, without limits in terms of status of researchers or specific diseases. Key questionnaire items relevant to building or describing research capacity were then extracted from these studies.

We then piloted these items on 24 investigators from TCM hospitals in Beijing. We interviewed participants using the questionnaire items which included questions such as “what do you think is the most important reflection of a clinical investigator's research capacity” and “what is the most helpful way to enhance one's research capacity?” We recorded their responses as a way of seeking suitable response options for each item.

Then we invited five specialists from different TCM research fields to provide further clarification. Amongst the specialists, there were two TCM clinical experts, one methodology expert, one editor of a TCM journal, and one policy maker in State Administration of Traditional Chinese Medicine. All were senior or associate directors and had worked for more than 15 years in their areas.

### 2.2. Questionnaire Items

The questionnaire items contained both open and closed questions organized in four domains: social demographic characteristics (5 items), academic and research characteristics (6 items), need for research capacity building (10 items), and barriers to clinical research (open question); see Appendix.

### 2.3. Recruitment and Questionnaire Distribution

All the participants were TCM clinical investigators attending a methodology training course organized by the National Administration of Traditional Chinese Medicine. Four rounds of training courses were held in Beijing between 2014 and 2015. Investigators from the 16 TCM clinical research centers in provinces of China were required to attend and were invited to participate in our survey. All were key staff in TCM hospitals or research centers.

Prior to the first day of each training course, members of our research team explained the purpose of the study and introduced the questionnaire. A paper version was then provided to every TCM investigator who was asked to complete it within 30 minutes, with assistance by a member of the research team if required. Responses were anonymous and were collected before the training session commenced. All the assistants were Ph.D. or graduate students of the EBM Centre in Beijing University of Chinese Medicine and had been trained before the questionnaires were distributed.

### 2.4. Analysis

Two members of our research team (FS and HM) entered the data we collected into Epidata 3.1. Consistency was checked in this software and corrected if inconsistent. The data was imported into SPSS (SPSS Inc., Beijing University of Chinese Medicine version 22.0). Descriptive analysis was undertaken for every item. In order to reflect the rank and scale of data, we aggregated the data and used frequency statistics to describe it. To analyze implicit factors such as the* number of publications *(first/corresponding author, that can be summarized as less than five/more than or equal to five), we used binary logistic regression. Covariates (*education*,* professional title*,* work experience,* and* status of being involved in project*) were tested for interaction as the model was built. If interaction existed, the association between publication and implicit factors was assessed by adjusted odds ratio (OR) with 95% confidence intervals (CI). For the covariates with more than 2 categories, the last category was considered as the reference group.

## 3. Results

### 3.1. Participation Rates

Of the 1280 questionnaires from 4 surveys in 2014-2015, 584 (45.63%) usable questionnaires were returned; the others were duplicated, incomplete, or unusable.

### 3.2. Social Demographic Characteristics

Social demographic characteristics of the participants are described in Table 1. Gender distribution of participants was almost equal (49.50% versus 50.50%), whilst the majority (43.15%) were 30 to 39 years of age. The majority of our participants (80.80%) held a minimum of a Master's degree qualification and the maximum part of them (40.24%) held a senior role/professional title. Just over half of our respondents (53.42%) reported working in an academic role for less than ten years.

### 3.3. Academic and Research Characteristics

The majority of investigators had directed a grant, whilst 16.78% of respondents had not had the opportunity to lead any kind of grant (see Table 2). A minority (12.50%) of participants had directed at least one national research grant. Most investigators (88.18%) had been involved in various levels of research projects and more than a half (55.48%) were members of national level projects. Most investigators (66.27%) reported reading less than 5 papers a month. Nearly half of investigators (48.80%) were first or corresponding authors of 1 to 5 publications, whilst the maximum part of them (42.11%) were coauthors on 1 to 5 publications. More than half had studied clinical epidemiology and medical statistics (54.11% and 56.51%, resp.). Only 30.14% of participants had studied EBM.

### 3.4. Relationship between Publication and Implicit Factors

As interaction existed between* work experience* and* professional title*, as well as* work experience* and* status of being involved in project*, odds ratio (OR) of implicit factors in the regression model was adjusted for* work experience*. Strong positive correlation was found between the number of publications and* number of professional articles read each month* (*p* < 0.001). Significant and positive correlations were also demonstrated for* education background* (*p* = 0.019),* professional title* (*p* = 0.025),* status of leading/being involved in project*, and* work experience* (*p* = 0.012). Those who had more publications had senior education degree and professional titles, longer work experience, and higher participation in research projects and in literature reading. However, the participation in methodology training did not influence the number of publications (*p* = 0.193); see Table 3.

### 3.5. Need for Research Capacity Building

**Figure 1:**
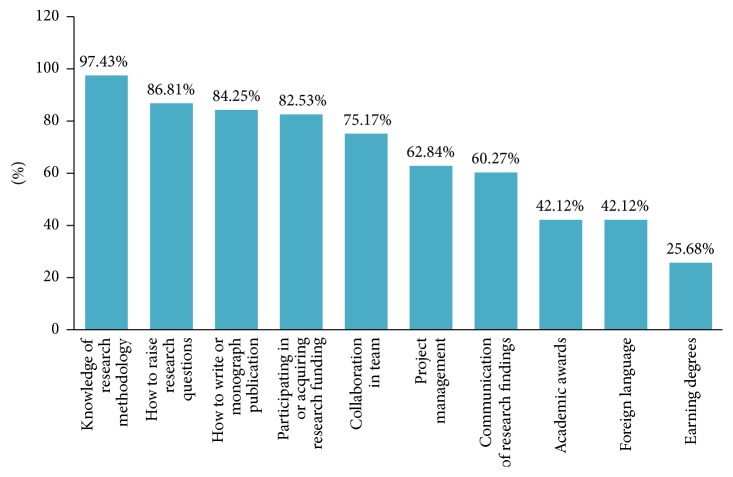
Need of research capacity building in TCM investigators.

The highest ranking research capacity need was* knowledge of research methodology* (97.43%), which was followed by* how to raise research questions* (86.81%),* how to write or monograph publication* (84.25%), and* participating in or acquiring research funding* (82.53%). Approximately eighty percent (80.13%) of participants had a demand for those choices. Other important needs identified were* collaboration in team* (75.17%),* project management* (62.84%), and* communication of research findings* (60.27%). The least important need identified was* earning degrees* which ranked last of all (25.68%) (see Figure 1).

### 3.6. Barriers to Clinical Research

**Figure 2:**
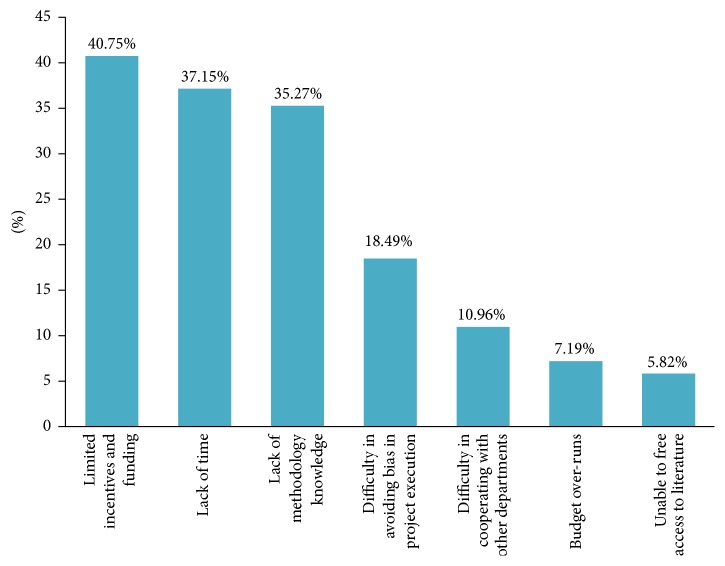
Barriers to clinical research for TCM investigators.

We used thematic analysis to analyze this open-ended question about barriers. The three most prominent problems were* limited incentives and funding* (40.75%),* lack of time* (37.15%), and* lack of methodology knowledge* (35.27%). Other barriers identified are related to the* difficulty in avoiding bias in project execution* (18.49%),* difficulty in cooperating with other departments* (10.96%),* budget over-runs* (7.19%), and being* unable to free access to literature* (5.82%); see Figure 2.

## 4. Discussion

Our findings suggest that most TCM investigators in China are below 40 years of age and have less than 10 years' experience in an academic role. Although many investigators were highly qualified academically and had received a doctorate, the vast majority of investigators were working at below a professor's grade.

Although the majority of our respondents reported they had the opportunity to conduct or assist in projects, our findings show a relatively low number of academic outputs amongst our respondents. Corresponding to this is that* how to write or monograph publication* was identified as one of the top ranking needs for building research capacity. Poor competency in English writing is an important factor. Poorly reported studies even if methodologically sound are not accepted by quality peer-review journals. Owing to the differences in philosophy and origin between TCM and conventional medical systems, investigators experience difficulties or delays in publishing TCM research, particularly in journals with high impact factor [14, 15]. Our findings also suggest that TCM investigators are not engaged with academic outputs: the majority of our respondents accessed fewer than 5 papers per month, which may diminish confidence in writing publication. This could be partly due to limited time, because most TCM investigators who hold practitioner positions in hospitals have an extremely large clinical workload and do not have the benefit of protected time for research [16, 17].

Our findings also indicate that* knowledge of research methodology* and raising research questions ought to be key priorities in developing research capacity. However, we acknowledge that our respondents were all attendees of a methodological training course and the results relating to need for methodological training may have been biased based on our sampling method. In China, research methodology courses are not compulsory for undergraduate TCM students and this may result in their weaker knowledge of research methodology. This partly explains the serious methodological deficiencies amongst Chinese-language publications, recently highlighted and criticized for blind pursuit of quantity without methodological quality [18–21]. Given the importance of methodological rigor in EBM and deficiencies within Chinese-language research, TCM investigators would welcome resources to support more investment in training and the benefit of protected time for research.

The highest ranking barrier to clinical research identified was* limited incentives and funding*. This is possibly related to a heavy clinical workload, which results in less time to prepare research grants. Poor methodological knowledge is another disadvantage in applying for funding. These factors resulted in fewer funded grants. Consistent with this, acquiring research funding was recognized as the fourth highest ranking research capacity need but it does also signal a need for an overall increase in funding for TCM research. In 2009, the Chinese government invested just 3 billion RMB (approximately $450,000) on healthcare research and development, whilst the US National Institutes of Health (NIH) spent in excess of $30 billion that year [22, 23]. This suggests an urgent need for increased investment in healthcare research by the Chinese government, particularly funds that are allocated specifically for TCM research, which would provide a significant boost in capacity and increase the opportunities available to TCM investigators.

*Limitations.* Due to limited resources, we were unable to pilot our questionnaire prior to the study. However, we employed other developmental methods to increase the quality of our instrument (literature review and consultation with experts). Another limitation was that respondents only had 30 minutes to complete the questionnaire and this time pressure may have influenced the responses provided; however, the questionnaire was relatively short and the closed questions reduced the burden on participants. Future research could include more qualitative research through in-depth interviews as a way of exploring complex views and perspectives about research capacity.

## 5. Conclusion

TCM clinical investigators in China generally had low-level of research capacity. Key priorities for building research capacity include methodological training, learning to formulate good research questions, and training to write research publications. Barriers include limited funding, lack of time, and shortcomings in methodological knowledge. Only investment in a sustainable research workforce will generate rigorous evidence on TCM treatments and practice. These areas need to be urgently addressed in order to improve the methodological quality of TCM researches and to ensure that TCM becomes increasingly evidence-based for clinicians, patients, and policymakers alike.

## Figures and Tables

**Table 1:** Social demographic characteristics of TCM clinical investigators.

Social demographic characteristics	*n*	%
Age (years)		
<30	150	25.68%
30–39	252	43.15%
40–49	132	22.60%
≧50	35	5.99%
Not provided	15	2.57%
Sex		
Male	289	49.50%
Female	295	50.50%
Education		
Doctor's degree	173	29.62%
Master's degree	299	51.20%
Bachelor's degree	106	18.15%
College degree	6	1.03%
Professional title/current role		
Professor	109	18.66%
Associate professor	126	21.58%
Intermediate researcher	160	27.40%
Assistant researcher	161	27.57%
other	28	4.79%
Work experience (years)		
<5	208	35.62%
5–9	104	17.81%
10–14	57	9.76%
15–19	57	9.76%
>20	132	22.60%
Not provided	26	4.45%

**Table 2:** Academic and research characteristics of TCM clinical investigators.

Academic and research characteristics	*n*	%
Status of directing a grant		
National level	73	12.50%
Provincial/ministerial level	169	28.94%
Municipal level	127	21.75%
Hospital/college level	107	18.32%
None	98	16.78%
Status of being involved in project		
National level	324	55.48%
Provincial/ministerial level	328	56.16%
Municipal level	161	27.57%
Hospital/college level	81	13.87%
None	69	11.82%
Number of professional articles read each month		
<1	179	30.65%
2–5	208	35.62%
6–10	116	19.86%
11–20	45	7.71%
>20	36	6.16%
Number of publications (first/corresponding author)		
0	61	10.45%
1–5	285	48.80%
6–10	108	18.49%
11–20	93	15.92%
>20	37	6.34%
Number of Publications (coauthor)		
0	117	20.08%
1–5	246	42.11%
6–10	105	17.93%
11–20	71	12.10%
>20	45	7.78%
Methodology Study		
Clinical Epidemiology	316	54.11%
Evidence-based Medicine	176	30.14%
Medical Statistics	330	56.51%

**Table 3:** Multivariate analysis of publications of TCM clinical investigators.

Implicit factors	*p* value	Odds ratio	% 95 CI
Education	0.019		
Doctor's degree	0.032	16.000	[1.267, 201.798]
Master's degree	0.426	2.543	[0.255, 25.327]
Bachelor's degree	0.423	2.630	[0.247, 28.047]
College degree	—	—	—
Professional title/current role	0.025		
Professor	0.026	2.480	[1.114, 5.519]
Associate professor	0.031	4.217	[1.136, 15.645]
Intermediate researcher	0.558	1.714	[0.283, 10.387]
Assistant researcher	—	—	—
Work experience (years)	0.012		
>20	0.028	2.809	[1.120, 7.048]
15–19	0.045	7.911	[1.046, 59.801]
10–14	0.082	3.697	[0.846, 16.168]
5–9	0.026	3.439	[1.163, 10.169]
<5	—	—	—
Status of being involved in project	0.045		
National level	0.998	4616953.670	—
Provincial/ministerial level	0.131	1.385	[0.908, 2.111]
Municipal level	0.994	22683641.660	—
Hospital/college level	0.004	2.473	[1.321, 4.496]
None	—	—	—
Number of professional articles read each month	0.000		
>20	0.998	807737421.426	—
11–20	0.0018	150008.500	—
6–10	0.000	45.000	[5.621, 360.287]
12–5	0.000	5.167	[2.353, 11.346]
<1	—	—	—
Methodology study	0.193		
Trained	0.193	1.539	[0.804, 2.947]
Not trained	—	—	—

## Appendix

### Questionnaire for Research Capacity of Clinical Researchers in National TCM Clinical Research Centers (English Version by Translation)

*Social Demographic Characteristics*

  Your gender
    □ Male
    □ Female
  Your age —
  What is the highest level of education you have achieved?
    □ Doctor's degree
    □ Master's degree
    □ Bachelor's degree
    □ College degree
  How many years of previous work experience you have got?
    □ <5 year
    □ 5–9 years
    □ 10–14 years
    □ 15–19 years
    □ >20 years
    □ Not provided
  What is your current role as a researcher?
    □ Full researcher/professor
    □ Associate researcher/professor
    □ Assistant researcher
    □ Research intern
    □ Other

*Academic and Research Characteristics*

  Have you ever been as the chief/sponsor of the following fund? (Please choose the highest level)
    □ National level research
    □ Provincial/ministerial level research
    □ Municipal level research
    □ Hospital/college level research
    □ None
  Have you ever participated in the following fund? (Please choose the highest level)
    □ National level research
    □ Provincial/ministerial level research
    □ Municipal level research
    □ Hospital/college level research
    □ None
  How many professional literature articles do you generally read in a month? —
  How many research article have you published as the first or corresponding author up to now? —
  How many research article have you published as the co-author up to now? —
  Have you had any of the following type of methodology training? (You may choose more than one option.)
    □ Clinical Epidemiology
    □ Evidence-based Medicine
    □ Medical Statistics
    □ None

*Need of Research Capacity Building*

  What is required in your research capacity build-up as a TCM clinical researcher? This question contains 10 choices (and the corresponding instructions) but 7 could be chosen at most.
    □ Knowledge build-up of research methodology (The necessary knowledge-base or skill about methodology, such as knowing the process about randomized allocation).
    □ How to raise research questions (The ability to think of good ideas for a research question for an upcoming grant application).
    □ Participating or acquiring research funding (Such as the ability of preparing biding document for funding applying).
    □ Project management (Using specialized skill, experience and method, to make the project excutive smoothly).
    □ Collaboration in team (Cooperate with the other members or departments in your team).
    □ How to write or monograph publication (The skill of writing papers or thesis).
    □ Foreign language (Such as English language competency in writing).
    □ Communication of research funding (Academic exchange or communication, such as the international conference, to share the research achievements).
    □ Earning degree (The funding application may contain a requirement of degree qualification to the applicants).
    □ Applying for prize for achievement (The outstanding research finding may indicate an upcoming prize, but also needs skills in achievements declaration).
  What do you think is the obvious barriers to restrict yourself in clinical research (open questions).
